# Electromagnetic Exposure Levels of Electric Vehicle Drive Motors to Passenger Wearing Cardiac Pacemakers

**DOI:** 10.3390/s24134395

**Published:** 2024-07-06

**Authors:** Xuwei Dong, Yidan Qian, Mai Lu

**Affiliations:** Key Laboratory of Opto-Electronic Technology and Intelligent Control of the Ministry of Education, Lanzhou Jiaotong University, Lanzhou 730070, China; 11220835@stu.lzjtu.edu.cn (Y.Q.); mai.lu@hotmail.com (M.L.)

**Keywords:** electric vehicles, cardiac pacemakers, electromagnetic exposure, drive motors

## Abstract

The number of individuals wearing cardiac pacemakers is gradually increasing as the population ages and cardiovascular disease becomes highly prevalent. The safety of pacemaker wearers is of significant concern because they must ensure that the device properly functions in various life scenarios. Electric vehicles have become one of the most frequently used travel tools due to the gradual promotion of low-carbon travel policies in various countries. The electromagnetic environment inside the vehicle is highly complex during driving due to the integration of numerous high-power electrical devices inside the vehicle. In order to ensure the safety of this group, the paper takes passengers wearing cardiac pacemakers as the object and the electric vehicle drive motors as the exposure source. Calculation models, with the vehicle body, human body, heart, and cardiac pacemaker, are built. The induced electric field, specific absorption rate, and temperature changes in the passenger’s body and heart are calculated by using the finite element method. Results show that the maximum value of the induced electric field of the passenger occurs at the ankle of the body, which is 60.3 mV/m. The value of the induced electric field of the heart is greater than that of the human trunk, and the maximum value (283 mV/m) is around the pacemaker electrode. The maximum specific absorption rate of the human body is 1.08 × 10^−6^ W/kg, and that of heart positioned near the electrode is 2.76 × 10^−5^ W/kg. In addition, the maximum temperature increases of the human torso, heart, and pacemaker are 0.16 × 10^−5^ °C, 0.4 × 10^−6^ °C, and 0.44 × 10^−6^ °C within 30 min, respectively. Accordingly, the induced electric field, specific absorption rate, and temperature rise in the human body and heart are less than the safety limits specified in the ICNIRP. The electric field intensity at the pacemaker electrode and the temperature rise of the pacemaker meet the requirements of the medical device standards of ICNIRP and ISO 14708-2. Consequently, the electromagnetic radiation from the motor operation in the electric vehicle does not pose a safety risk to the health of passengers wearing cardiac pacemakers in this paper. This study also contributes to advancing research on the electromagnetic environment of electric vehicles and provides guidance for ensuring the safe travel of individuals wearing cardiac pacemakers.

## 1. Introduction

With increasing global climate change and energy security problems, the exhaust emissions from traditional vehicles have caused significant pollution to the environment. Electric vehicles (EVs), a representative of low-carbon transportation, can effectively reduce exhaust emissions, reduce air pollution, and have a positive influence on the suppression of global climate change, and the importance of EV in the future transportation system cannot be ignored [1]. Therefore, countries have introduced encouraging policies in recent years to promote the rapid development of EV and improve the level of energy saving and emission reduction [2].

The increasing popularity of EVs has raised widespread concern about the safety of the electromagnetic environment. Gryz et al. [3] studied the electromagnetic environment inside an EV in urban traffic. The results show that the electromagnetic fields inside the vehicle meet the safety limits in the guidelines for the assessment of human exposure provided by the International Commission on Non-Ionizing Radiation Protection (ICNIRP), the European Parliament, and the Council of the European Union. Fotescu et al. [4] compared the electromagnetic field and electric field radiation inside EVs and fuel vehicles. The results demonstrated that the radiation values of EVs were higher than those of fuel vehicles on different roads. Yang et al. [5] found, in a long-term monitoring study of EVs, that the acceleration of vehicle will increase the intensity of the magnetic field inside the vehicle, and the replacement of vehicle components will also affect the magnetic field in the vehicle. Some studies had also measured the electromagnetic radiation level of different seating positions in EVs and compared them with electromagnetic radiation standards; the measurement results meet the standard requirements [6,7,8]. Meanwhile, extensive research has been undertaken on the electromagnetic environment for EV wireless charging. The results [9,10,11] showed that the measured values of the internal and external electromagnetic environments of the EV during wireless charging were lower than the limit specified by ICNIRP [12].

In addition to the study of the electromagnetic environment inside and outside the EV, the interaction between the electromagnetic environment and the human body has also received significant attention. Xiao et al. [13] established the radiation model of the DC converter in the EV and calculated the distribution of the specific absorption rate (SAR) in the passenger’s body. Hakuta et al. [14] proposed the coupling factor method and calculated the induced electric field inside the passenger body under the influence of the electromagnetic environment generated by the power cable of the EV. Wang et al. [15] calculated the electromagnetic field distribution inside the EV with the electromagnetic calculation software, and the SARs in the passenger’s body at different positions were also calculated. Concha et al. [16] simulated the magnetic field generated by a single battery with the finite element method, and then estimated the impact of the magnetic field generated by a complete battery pack on the electromagnetic exposure of passengers. Dong et al. [17] studied the electromagnetic environment generated by the inverter of the EV and calculated the distribution of inductive fields in the driver’s different tissues and organs. Tan et al. [18] studied the electromagnetic exposure and protection caused by low-frequency electromagnetic fields in the EV under different operating conditions. Benni and Toknola et al. [19,20] studied the electromagnetic radiation effects of automobile communication antennas on passengers and pedestrians and compared the results with the ICNIRP guidelines to evaluate the electromagnetic exposure safety of the communication antennas to the human body. Zhao et al. [21] calculated the induced electric field in the human body during the charging of electric vehicles under different wireless charging conditions and the results are compared with the ICNIRP to derive the minimum safe distance for the human body.

In 2023, the “Global Burden of CVD Disease Report” published by the Journal of the American College of Cardiology [22] highlighted the increasing global death toll attributed to cardiovascular disease, indicating a growing population reliant on implantable medical devices to maintain a normal life [23]. Although implantable medical devices can improve the quality of life of the wearer, the potential influence of external electromagnetic fields on these devices has been a matter of serious concern for clinicians and patients. Studies have shown that passengers with brain pacemakers have experienced dizziness, heart palpitations and other symptoms when riding and driving Toyota Prius hybrid EV, which may be related to the electromagnetic field inside the vehicle [24]. In addition, relevant studies have shown that electromagnetic fields generated by electronic devices may be misperceived by cardiac implantable electronic devices, causing inhibition of pacing and threatening the lives of patients [25,26]. However, the electromagnetic radiation generated by EVs may also affect the normal function of pacemakers in individuals who wear them [27,28], thus posing a non-negligible threat to patients. Tian et al. [29] studied the impact of electromagnetic environment generated by high-speed train pantograph on the safety of passengers wearing cardiac pacemakers in different seating positions. The calculated results were compared with the limits specified by the ICNIRP and the International Organization for Standardization (ISO) to evaluate the safety of the electromagnetic environment for these special passengers. Wang et al. [30] found that when passengers implanted with coronary artery stents were near the wireless charging system of EVs, the induced electric field intensity in their body would increase, resulting in certain security risks. Some studies were also conducted regarding the influence of electromagnetic environment generated by wireless charging process on pacemaker wearers to alleviate their anxiety [31,32]. Although the wireless charging system did not affect the normal operation of the pacemaker, the SAR and temperature of the human tissue around the pacemaker will significantly increase when the pacemaker is in close proximity to the radiation source [33,34].

Therefore, there were many studies mainly focused on the electromagnetic exposure level of passengers wearing pacemakers caused by wireless charging systems, while the impact of electromagnetic environment generated by the drive motor of EVs for the safety of such passengers has not been carried out. The drive motor is a vital component of the EV drive system, acting as the direct power source of the vehicle. The experimental results regarding electromagnetic interference in the EV drive system showed that the electromagnetic interference level was increased in the case of high speed and large torque of the motor [35]. During EV driving, especially in the acceleration and braking process, the frequently changing current inside the motor will generate a high-frequency spatial electromagnetic field around it [36,37,38]. Therefore, in order to evaluate the influence of high-frequency electromagnetic fields generated by the driving motors of the EV on the health of passengers wearing cardiac pacemakers, the finite element calculation software Comsol Multiphysics 6.0 is used to analyze the electromagnetic exposure level of passengers wearing cardiac pacemakers in the four-wheel drive vehicle with dual motors as the radiation source in this paper. The induced electric field (E_in_), specific absorption rate (SAR) and temperature change in the passenger’s body are calculated. The numerical calculation results are compared with the safety limits specified by relevant standards to evaluate the safety of the electromagnetic environment generated by the EV drive motor for the pacemaker wearer. The study will advance research on the electromagnetic environment of EV, eliminate the concerns of passengers wearing cardiac pacemakers about the safety of electromagnetic exposure in EV to a certain extent and provide suggestions and guidance for their safe travel.

## 2. Numerical Calculation Methods and Model Construction

### 2.1. Numerical Calculation Method

The radio frequency (RF) electromagnetic wave module (RF module) is used to calculate and analyze the electromagnetic field in Comsol Multiphysics 6.0 software. The module is based on the phasor form of Maxwell’s equations in a time-varying electromagnetic field:

(1) ∇×H=J+jωD

(2) ∇×E=−jωB

(3) ∇·B=0

(4) ∇·D=ρ

The wave equation in the frequency domain of electromagnetic wave is obtained by combining the above formulas:

(5) $$\nabla \times {\mu_{r}}^{-1}\left(\nabla \times E\right)-{k_{0}}^{2}\left(\varepsilon_{r}-\frac{j\sigma }{\omega \varepsilon_{0}}\right)E=0$$

where *j* is the imaginary unit, ω is the angular frequency, *B* is the magnetic flux density, *H* is the magnetic field intensity, *J* is the current density, *D* is the electrical displacement, $\mu_{r}$ is the relative permeability, $\varepsilon_{r}$ is the relative permittivity, $\varepsilon_{0}$ is the relative permittivity of the vacuum, σ is the conductivity, and the electric field intensity can be obtained.

According to the International Guidelines for Electromagnetic Radiation Exposure (ICNIRP-2020) [39], the SAR is used to determine the amount of radiation absorbed by human tissues under the influence of electromagnetic radiation from 100 kHz to 6 GHz. However, given that the SAR values in the body and organs cannot be directly measured in the human body, the numerical calculation method is used in this paper to calculate the SAR in passenger’s body, and the calculation formula is as follows:

(6) $$\mathrm{SAR}=\frac{\sigma {|E|}^{2}}{\rho }$$

where σ is the conductivity in S/m, and *E* is the induced electric field intensity in V/m.

In addition, due to the temperature increase caused by the absorption of electromagnetic radiation in biological tissues, the electromagnetic and temperature fields are coupled in this paper. The temperature rise of the human body, heart, and pacemaker are calculated. Fourier’s thermal conductivity law and Pennes bioheat transfer equation [40] are mainly used in the calculation:

(7) $$q=-k\frac{dT}{d\tau }$$

(8) $$\rho c_{p}\frac{\partial T}{\partial t}+\nabla \cdot \left(q\right)=\rho \cdot SAR+\rho_{b}c_{p}w_{b}\left(T_{b}-dT\right)+Q_{met}$$

where ρ is the material density in kg/$m^{3}$, *k* is the thermal conductivity of the material in W/$m\cdot K,\;\;w_{b}$ is the blood perfusion rate in 1/s, $c_{p}$ is the specific heat capacity of blood in J/kg$\cdot K,\;\;T_{b}$ is animal blood temperature in K, $Q_{met}$ is a metabolic heat source in J/$m^{3}\cdot s$.

### 2.2. Electric Vehicle Body Model

**Figure 1:**
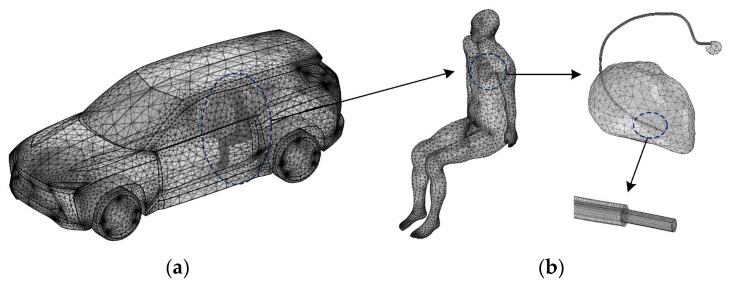
Mesh grid models: (**a**) vehicle body mesh model; (**b**) passenger body with cardiac pacemaker mesh model.

The vehicle model is a medium to large EV, the body material is aluminum alloy, and the four-wheel drive design is adopted. According to the real vehicle body size, the EV body model with 5099 mm × 1989 mm × 1750 mm is built by using 3D modelling software (Solidworks 2020). Some parts with complex shapes and difficult modelling, such as seats, mirrors, and headlights, are removed during the model construction to simplify the 3D physical model, minimize the distortion generated in the finite element mesh subdivision, and shorten the model calculation time. After the simplification of the vehicle body model, a tetrahedral mesh is used to divide the vehicle body and the passenger. The number of mesh grids of the vehicle body and the passenger are 320,775 and 526,228, respectively. For the mesh model of the cardiac pacemaker, due to the small size of the lead and the insulation layer, the custom grid is used to divide the lead and insulation layer. The mesh grids resolution is 0.2 mm × 0.2 mm × 0.2 mm. The mesh grid of the electric vehicle body and passenger body with cardiac pacemaker is shown in Figure 1.

### 2.3. Passenger Model Wearing a Pacemaker

#### 2.3.1. Human Body Model and Related Parameters

The human body model in this paper is a real digital human body model constructed based on high-resolution magnetic resonance images (MRIs) and derived from Duke, a 34-year-old adult male model in the Virtual Family Project [41]. MRI can improve the soft tissue contrast and is more suitable for identifying and segmenting different body tissues and organs compared with computed tomography (CT) imaging. During the calculation, the dielectric parameters (permittivity and conductivity) of human tissues are obtained based on the fourth-order Cole–Cole model [42]. Specifically, the dielectric parameters of the human torso are taken as the average of the four tissues of skin, blood, muscle, and bone. Physical quantities, such as tissue density, specific heat capacity, thermal conductivity, and blood perfusion rate, that relate to the temperature rise of the human body are shown in Table 1.

#### 2.3.2. Pacemaker Model

**Figure 2:**
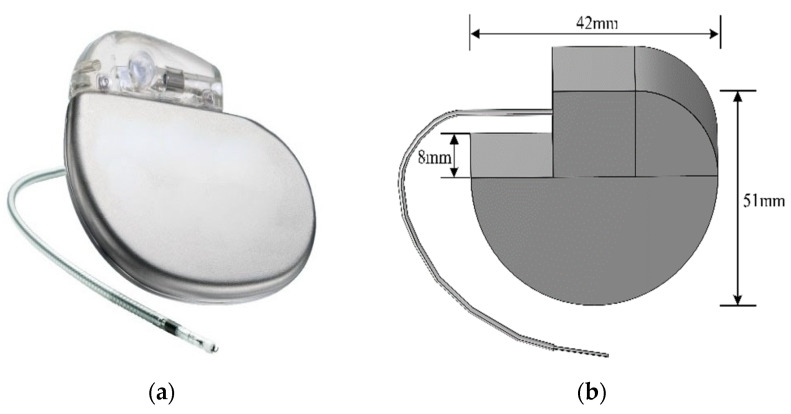
Schematic of a cardiac pacemaker: (**a**) unipolar pacemaker; (**b**) physical model of a pacemaker.

**Figure 3:**
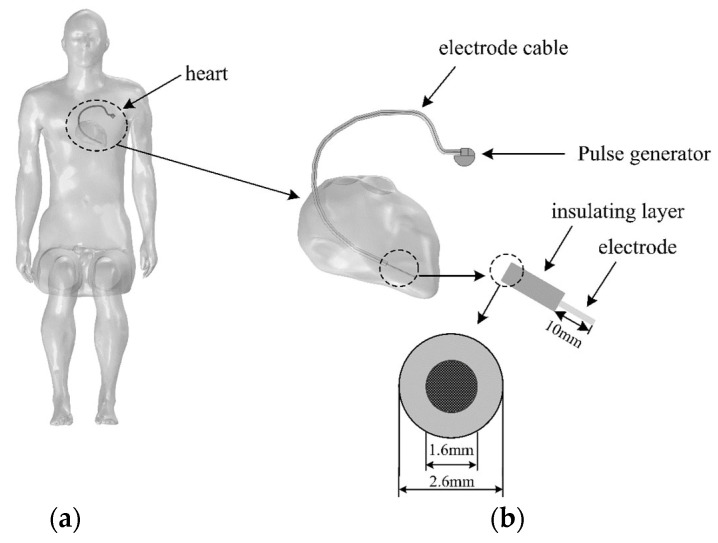
Passenger model with a pacemaker: (**a**) human sitting posture model; (**b**) heart and pacemaker model.

The pacemaker model is based on a specific type of unipolar pacemaker (Figure 2a). The pacemaker could help patients with heart disease recover their physiological heart rate, improve cardiac output, and prevent disease or arrhythmias caused by heart beat formation or conduction disorders. In this paper, a pacemaker model is constructed according to its real size and material, which includes a pulse generator and an electrode lead. The pacemaker model built with modelling software is shown in Figure 2b, and its parameters are shown in Table 2. The pacemaker electrode material is platinum-iridium alloy with a diameter of 1.4 mm and the insulation layer material is silicone rubber with a diameter of 2.6 mm. The 10 mm insulation layer is removed at the tip of the electrode and the tip is inserted into the right ventricle of the heart to contact the myocardial tissue. Figure 3 shows the entire model of the wearer and pacemaker after the device is implanted in the human body.

### 2.4. Electromagnetic Environment Model

The drive motor, an important energy conversion device of EV, converts electrical energy into mechanical energy to drive the vehicle. However, due to the complexity of the physical and circuit structure of the driving motor, if the drive motor model is built strictly according to the motor principle and mathematical model, it will not only be complex, but also increase the complexity of the finite element calculation, consume a substantial amount of hardware resources, and result in large calculation errors. Accordingly, the drive motor will generate electromagnetic fields in the space when it works and affect the environment around the drive motor in the form of electromagnetic radiation. In this paper, combined with the antenna radiation principle [43,44], the complex circuit structure of the driving motor is equivalent to a dipole antenna model. Given that the radiation mode of the dipole antenna depends on the antenna length and the frequency used, the length of the antenna is set as half wavelength of the electromagnetic wave in this work. The operating wavelength of the antenna is 3.75 m, the dipole arm of the antenna is 1/4 of the wavelength, the radius of the dipole antenna arm is 1/20 of the wavelength, and the gap between the two arms is 1/100 of the wavelength. A lumped port is set between the dipole arms and an exciting voltage of 0.3 V is applied. The space field generated during the operation of the drive motor is equivalently simulated by the electromagnetic field induced on the adjacent conductive surface.

**Figure 4:**
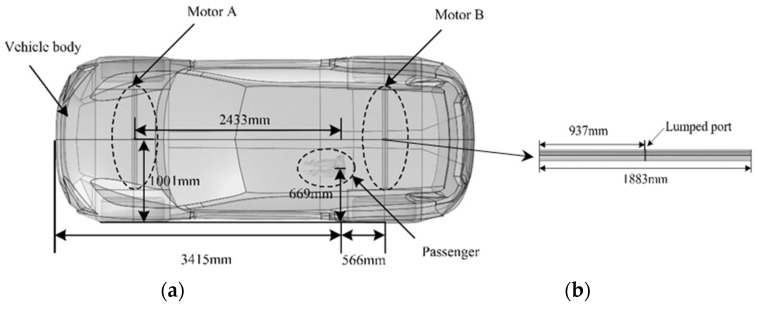
Electromagnetic environment model of EV: (**a**) relative position of the passenger and the drive motor equivalent models; (**b**) drive motor equivalent model.

In addition, there were studies had calculated with equivalent source modelling method and measured the spatial electromagnetic environment generated by the drive motor with a maximum speed of 6000 r/min in an EV that found that the spatial field inside the vehicle reached the maximum near 80 MHz [15,45]. Accordingly, an equivalent source of the dipole antenna model is built in Comsol Multiphysics software to study the influence of the spatial field generated by the drive motor of the EV on the passenger wearing cardiac pacemaker. The distribution of different induction fields inside the body of the passenger wearing cardiac pacemakers is also calculated. The electromagnetic environment model of EV in this paper is shown in Figure 4.

## 3. Analysis of the Calculation Results

In this study, the multi-physical field coupling method is used for calculation. The high-frequency electromagnetic field generated during the operation of the drive motor is coupled with the heat transfer of biological tissues and cardiac pacemakers, and the electromagnetic heat is solved. The calculated results are compared with the safety restrictions defined by the ICNIRP and the international standards for medical devices. The safety of electromagnetic exposure of passengers wearing cardiac pacemakers in the high-frequency electromagnetic environment generated by drive motors of EV is evaluated.

### 3.1. Distribution of Electric Field Intensity in the Carriage

**Figure 5:**
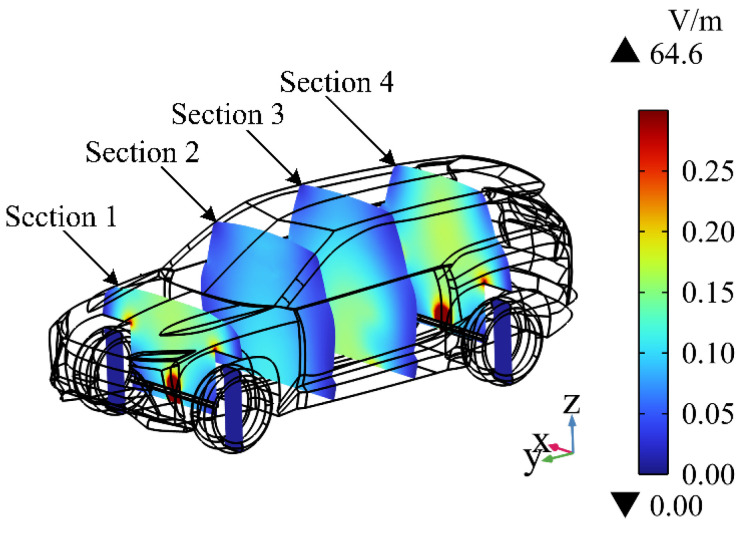
Cross-section of the electric field intensity at different positions in the carriage.

Four sections (Section 1, Section 2, Section 3 and Section 4) parallel to the XZ plane are selected in the carriage to study the distribution of the electric field intensity in the carriage of the EV. As shown in Figure 5, Section 1 and Section 4 are tangent to the center of the radiation source; these two sections have the largest electric field intensity, with the maximum value of 64.6 V/m. The electric field intensity gradually decreases with the increase in the distance from the drive motor. Section 3 is located at the same central position from the front and rear motors, and the electric field is superimposed. The electric field intensity is larger than that on Section 2, with the maximum value of 0.12 V/m.

### 3.2. Distribution of Induced Electric Field in the Passenger’s Body

**Figure 6:**
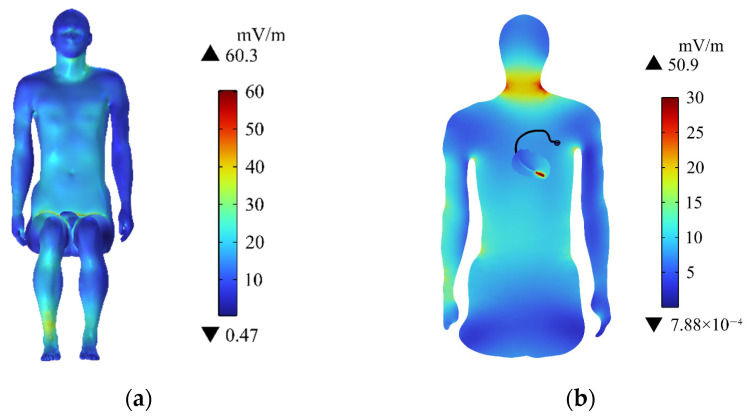
Distributions of the E_in_: (**a**) E_in_ on the human body surface; (**b**) E_in_ in the central section of the human upper limb.

The distribution of the induced electric field (E_in_) in the passenger’s human body surface is shown in Figure 6a. E_in_ is larger in the neck, right arm, chest and abdomen, and calf, whilst the other parts have smaller values on the passenger body surface. The maximum value of E_in_ (60.3 mV/m) appears in the passenger’s right calf. This situation is related to the distance from the human body to the radiation source. The closer the distance, the greater the E_in_. The passenger’s right calf is the closest to the motor. Hence, E_in_ is the largest.

Figure 6b shows the distribution of E_in_ on the central section of the human upper limb. E_in_ near the pacemaker electrode in the passenger’s heart is the largest (50.9 mV/m). Meanwhile, E_in_ in the passenger’s neck region is also relatively large, but it is less than the value near the pacemaker electrode. E_in_ in the rest of the upper limb is much smaller than that of the passenger’s neck. The maximum of E_in_ in the passenger’s body does not exceed the public exposure limit (27.7 V/m) of the electromagnetic field defined by ICNIRP [39].

### 3.3. Distribution of Induced Electric Field in the Heart

**Figure 7:**
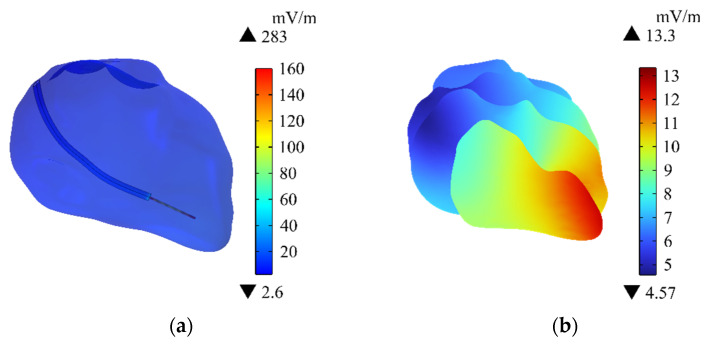
Distributions of E_in_: (**a**) E_in_ in the heart; (**b**) E_in_ in different longitudinal sections of the heart.

Figure 7a shows the distribution of E_in_ in the heart. E_in_ on the surface of the heart is small. Furthermore, E_in_ gradually increases as the pacemaker electrode gets closer and reaches the maximum value (283 mV/m) near the pacemaker electrode, which is lower than the public exposure safety limit specified by ICNIRP [39]. E_in_ on the different longitudinal sections of the heart is shown in Figure 7b to better understand the distribution of E_in_ inside the heart. The region with larger E_in_ is mainly concentrated at the tip of the heart, and the closer to the pacemaker electrode, the larger the E_in_. This is related to the electrical conductivity and permittivity of pacemaker electrodes. The pacemaker electrode is commonly made of platinum–iridium alloy, whose conductivity is high. Accordingly, E_in_ near the pacemaker electrode is larger than those in other areas of the heart and the surface of the human body. Although E_in_ near the pacemaker electrode is high, the electric field level does not exceed the safety limit of 28 V/m specified by ICNIRP [46].

### 3.4. Specific Absorption Rate of Human Body

**Figure 8:**
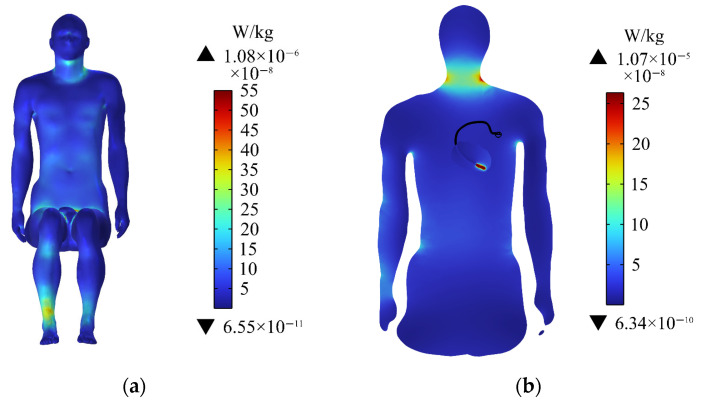
Distributions to the SAR: (**a**) SAR on the human surface; (**b**) SAR in the central section of the human upper limb.

The distribution of SAR on the human body surface is shown in Figure 8a. The SAR of the neck and calf area of the human body is larger, and the maximum value is at the right calf, which is 1.08 × 10^−6^ W/kg. Figure 8b shows the distribution of the SAR on the central section of the human upper limb. The SAR near the neck of the passenger and the pacemaker electrode is relatively large. The maximum SAR near the neck is 2.64 × 10^−7^ W/kg, and that near the pacemaker electrode is 1.07 × 10^−5^ W/kg. The average SAR value of the passenger’s whole body is 2.91 × 10^−8^ W/kg. The ICNIRP [39] guideline requires that the average SAR of the whole body does not exceed 8 × 10^−2^ W/kg. Thus, the SAR of the human body in this exposure scenario is far below the safety limit specified in ICNIRP.

### 3.5. Specific Absorption Rate of Heart

**Figure 9:**
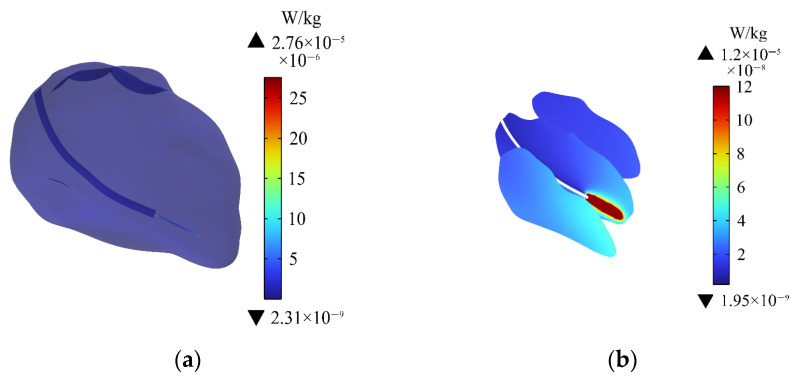
Distributions of the SAR: (**a**) SAR in the heart; (**b**) SAR on the different longitudinal sections of the heart.

Figure 9a shows the distribution of SAR in the heart of the passenger. The distribution of SAR is similar to the distribution of E_in_ in the heart, and the maximum value (2.76 × 10^−5^ W/kg) is still around the pacemaker electrode. Figure 9b shows the distribution of SAR on the different longitudinal sections of the heart. The closer to the pacemaker electrode, the greater the SAR is. Although the SAR in the heart is larger than the average SAR of the entire body of the passenger, the SAR in the heart is still within the exposure safety limit stipulated by the ICNIRP [39].

### 3.6. Temperature Rise of the Human Body

After absorbing electromagnetic waves, the biological tissues will convert electromagnetic energy into heat energy, which will produce a biological thermal effect and increase the temperature of the biological tissues. ICNIRP [39] is used as a reference to determine the principle of human tissue temperature rise threshold. This paper calculates the temperature rise of the human body and the heart within 30 min and compares with the temperature rise threshold specified in ICNIRP [39].

**Figure 10:**
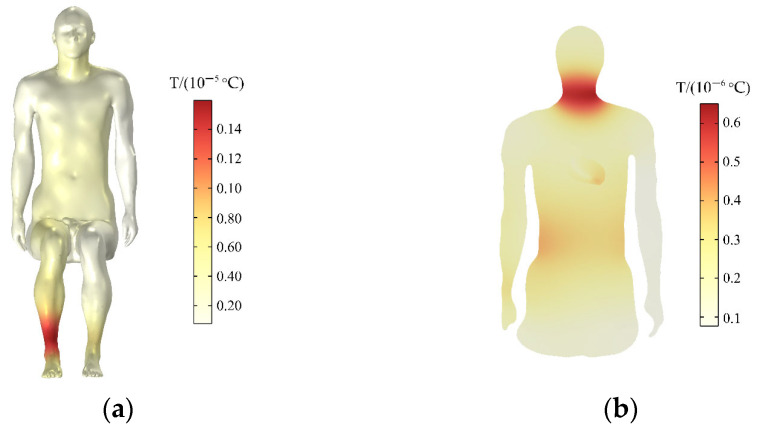
Distributions of the temperature rise: (**a**) temperature rise of the human body; (**b**) temperature rise in the central section of the human upper limb.

The initial temperature of the human tissue is 36.4 °C. The temperature rise of the passenger’s body is shown in Figure 10a. Meanwhile, the temperature of the entire body is increased by a maximum of 0.16 × 10^−5^ °C within 30 min, the temperature rise is greatest in the right calf. The temperature rise on the rest surface of the human trunk is relatively uniform. Figure 10b shows the temperature rise in the central section of the human upper limb. The temperature rise on the human neck in this section is greater than that of the heart within 30 min, and the maximum temperature rise on the neck is 0.6 × 10^−6^ °C.

### 3.7. Temperature Rise of the Heart

**Figure 11:**
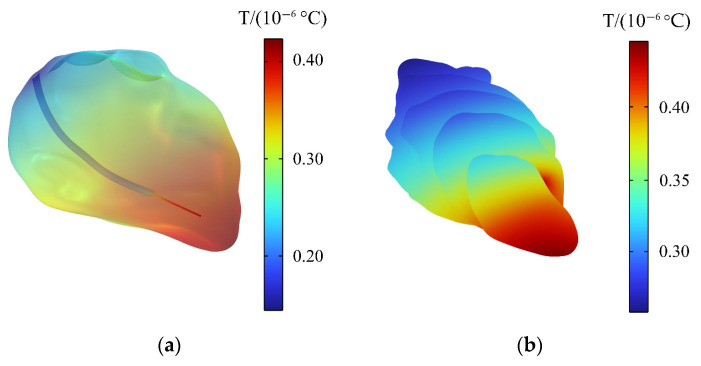
Distributions of the temperature rise: (**a**) temperature rise of the heart; (**b**) temperature rise on longitudinal sections of the heart.

The temperature rise of the heart is shown in Figure 11a. The temperature of the heart is raised by 0.4 × 10^−6^ °C within 30 min, and the maximum temperature rise of the heart does not exceed the temperature rise limit (1 °C) of the thermal injury stipulated by ICNIRP [39]. Accordingly, the normal physiological activities of the human body would not be affected. Figure 11b shows the distribution of temperature rise on the different longitudinal sections of the heart. The temperature increases the most near the tip of the heart, and the farther away from the tip, the smaller the temperature increase.

### 3.8. Temperature Rise of the Pacemaker

**Figure 12:**
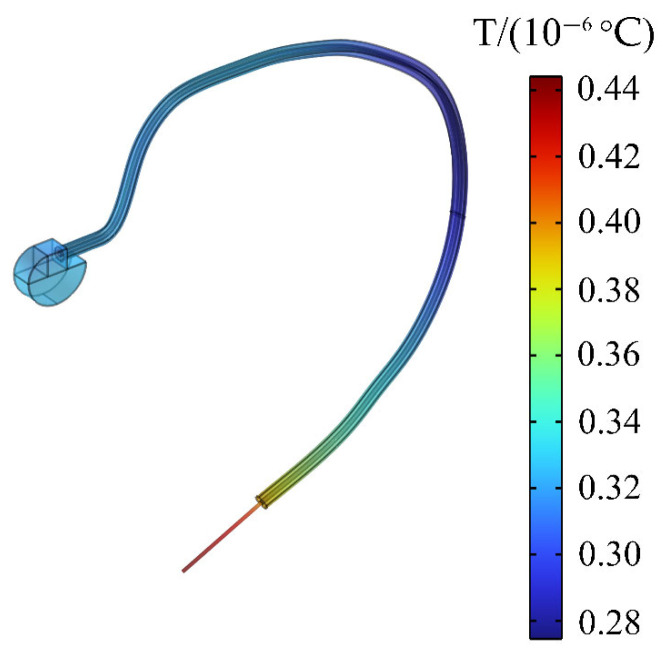
Temperature change of the pacemaker.

When a passenger wearing a pacemaker is in a high-frequency electromagnetic environment, the implanted pacemaker will exchange heat with the surrounding tissues through heat conduction. The temperature change of the pacemaker within 30 min is also calculated in this paper to analyze the influence of the thermal effect generated by high-frequency electromagnetic radiation on the pacemaker. In Figure 11a, it can be clearly seen that the pacemaker electrode head has a slightly higher temperature rise than the heart. Figure 12 shows that the maximum temperature rise in the pacemaker is on the pacemaker tip, with a maximum temperature rise of 0.44 × 10^−6^ °C. The temperature rise also meets the requirement of ISO 14708-2 that the temperature rise of medical equipment does not exceed 2 °C [47]. Therefore, the high-frequency electromagnetic environment generated by the drive motor will not affect the normal operation of the pacemaker.

## 4. Discussion

With the world’s attention turning to environmental protection and sustainable transportation, the development of electric vehicles is rapid. High levels of electrification and intelligence are becoming the trends and major research topics of future automotive technology development. However, unlike traditional fuel vehicles, EVs exhibit a highly complex electrical system. The drive motor, a key component of electric energy conversion into mechanical energy in EV, will generate high-frequency transient pulse current and cause high-frequency electromagnetic radiation. This situation may have a little influence on ordinary passengers. Nonetheless, high-frequency electromagnetic radiation is likely to affect the normal function of the cardiac pacemaker and threaten the life safety of these special passengers wearing cardiac pacemakers. Accordingly, whether patients can safely ride or approach these vehicles whilst wearing cardiac pacemakers is evaluated to study the influence of high-frequency electromagnetic radiation generated by drive motors of EV on passengers wearing pacemakers and the function of cardiac pacemakers, and relevant preventive measures and travel recommendations are provided. In this paper, the driving motor of EV is the exposure source. The influence of the driving motor on the passenger wearing cardiac pacemaker is studied with a radiation frequency of 80 MHz.

The distribution of the spatial field inside the EV and the E_in_, SAR, and temperature in the body of the passenger wearing cardiac pacemaker are calculated by using the finite element method. The maximum E_in_ on the human body surface is around the calf, and that in the heart is around the pacemaker electrode; the maximum values are 60.3 mV/m and 283 mV/m, respectively. By contrast, E_in_ in the passenger’s body and heart does not exceed the public exposure limits for electromagnetic fields specified by ICNIRP [39]. The maximum SAR of the human body and heart are 1.08 × 10^−6^ W/kg and 2.76 × 10^−5^ W/kg, respectively. Given the positive correlation between SAR and E_in_, the maximum distribution of SAR is similar to that of E_in_, and the SAR is also within the public exposure limits stipulated by ICNIRP [39]. In addition, the temperature rise of the human body and the heart with 30 min is calculated according to the determination method of the safe limit of the human temperature rise specified by ICNIRP [39]. The temperature of the passenger’s whole body is increased by a maximum of 0.16 × 10^−5^ °C within 30 min, and the temperature rise of the passenger’s body is relatively uniform except for the right calf. The passenger’s heart temperature is increased by a maximum of 0.4 × 10^−6^ °C, and the maximum value occurs on the outer surface of the heart. The temperature rise of the passenger’s body and heart is also less than the safety threshold specified by ICNIRP [39]. In addition, the maximum temperature rise of the pacemaker tip is 0.44 × 10^−6^ °C, which also meets the requirement of the ISO 14708-2 specified by International Organization for Standardization that the temperature rise of medical equipment does not exceed 2 °C.

## 5. Conclusions

By analyzing the distribution of induction field and temperature rise of the passenger wearing cardiac pacemakers, the following conclusions can be drawn: Implantation of cardiac pacemakers will have an impact on the field distribution around the heart of the passenger, but by comparing with the safety limits stipulated by the ICNIRP and ISO, the high-frequency electromagnetic radiation generated by the drive motor in this paper would neither affect the health of the passenger wearing the cardiac pacemaker nor does it pose a threat to the normal function of the cardiac pacemaker. The results of this study can eliminate the concerns of passengers wearing cardiac pacemakers on the electromagnetic environment safety of EV a certain extent and provide suggestions and guidance for their safe travel. Moreover, the results contribute to advancing research on the electromagnetic environment of EV and provide references for the revision of electromagnetic exposure standards for EV and the design of electromagnetic exposure safety protection for vehicles.

## Figures and Tables

**Table 1:** Relevant parameters of human tissues (f = 80 MHz).

Tissue	σ (S/m)	ε	ρ $\left(kg/m^{3}\right)$	*C* W/(kg·°C)	k W/(m·°C)	$\omega_{b}$ 1/s
skin	0.4843	76.1495	1109	3391	0.37	0.0016
blood	1.2196	81.0520	1050	3617	0.52	—
muscle	0.6977	68.7690	1090	3421	0.49	0.00056
Bone	0.1783	27.6897	1908	1313	0.32	0.000087
heart	0.7049	97.9640	1059	1.22	0.43	0.02

**Table 2:** Material and geometric parameters of a cardiac pacemaker.

Cardiac Pacemaker Parameters	Material or Size
Cardiac pacemaker casing	Titanium
Electrode conductor	Platinum-iridium alloy
Insulating layer	Silicone rubber
Connector module	75D polyurethane
Geometric parameters (H × W × D)	51 mm × 42 mm × 8 mm
Surface area	$32.2{\;\mathrm{cm}}^{2}$
Quality	21 g

## Data Availability

The data that support the findings of this study are available from the corresponding author upon reasonable request. The data are not publicly available due to privacy.
